# Noninvasive assessment of hemodynamic profile and myocardial mechanics in pulsus alternans patients by multiple echocardiographic methods

**DOI:** 10.1186/s13089-025-00448-y

**Published:** 2025-10-06

**Authors:** Rongrong Miao, Tingting Wu, Qilong Wu, Teng Ye, Hui Li, Shusheng Liao

**Affiliations:** 1 Department of Ultrasound, The First Affiliated Hospital of Wenzhou Medical University, Wenzhou, 325000 Zhejiang China; 2 Department of Operating Room, The First Affiliated Hospital of Wenzhou Medical University, Wenzhou, Zhejiang China

**Keywords:** Pulsus alternans, Left ventricular, Systolic, Echocardiography, Strain

## Abstract

**Background:**

Pulsus alternans (PA) is an intriguing phenomenon and a clinically rare entity. Accurately assessing cardiac function in patients with PA remains challenging. This study aims to investigate the myocardial mechanical characteristics and non-invasive hemodynamic profiles of PA patients using multiple echocardiographic imaging modalities.

**Methods:**

Clinical and echocardiographic data were retrospectively analysed from 16 patients diagnosed with PA by echocardiography at our hospital between January 2021 and May 2025. In this study, the characteristics of PA were elaborated by multiple echocardiographic methods, and the non-invasive hemodynamic profile was determined by pulse-wave Doppler.

**Results:**

Sixteen patients were enrolled. Seven were classified as NYHA class III and six as class IV. Elevated levels of NT-proBNP and hs-cTNT were observed in most patients. Follow-up ranged from 1 to 44 months, and five patients experienced adverse outcomes, including heart transplantation, rehospitalisation, and death. Within this cohort, three patients exhibited biventricular PA, while 13 patients presented with left ventricular (LV) PA. Key hemodynamic parameters varied significantly: LVOT-VTI_strong beat_ ranged from 11.3 cm to 29.2 cm, LVOT-VTI_weak beat_ from 6.8 cm to 22.1 cm, and the variation rate between strong and weak beats (∆LVOT-VTI) ranged from 19 to 52%. Global longitudinal strain (GLS) was significantly reduced in 14 patients (range: − 1.2% to − 10.4%), while peak strain dispersion (PSD) increased (range: 47 ms to 117.5 ms). Two patients were excluded from strain analysis due to suboptimal imaging. Hemodynamic parameters (LVOT-VTI_strong beat_, LVOT-VTI_weak beat_ and ∆LVOT-VTI) showed strong correlations with GLS in PA patients (*r* = 0.806, *P* = 0.001; *r* = 0.642, *P* = 0.018 and *r* = 0.611, *P* = 0.027, respectively). NT-proBNP was significantly positively related to adverse outcomes in PA patients (*r* = 0.669, *P* = 0.012).

**Conclusion:**

Echocardiography is essential for evaluating cardiac function in patients with PA. This study used multiple echocardiographic methods to delineate the characteristics of this intriguing clinical phenomenon. Non-invasive hemodynamic parameters are potentially important for prognosis assessment, and myocardial strain assessment provides valuable insights into myocardial mechanical features. A comprehensive analysis using multimodality imaging is crucial for accurately identifying this disease, potentially enhancing the understanding of the pathophysiological mechanism of PA.

## Background

Since Traube [1] first described pulsus alternans (PA) in 1872, its definition has expanded to encompass a spectrum of physiological and pathological phenomena. However, patients with cardiac alternans exhibit significant variations in aetiology, mechanism, and clinical implications. Cardiac alternans can be classified into four types: (1)mechanical, (2)electrical, (3)correlated with myocardial ischemia, and (4) correlated with cardiac motion [2]. Mechanical alternans refers to alternating strong and weak ventricular contractions despite a regular heart rhythm, manifesting as left ventricular (LV) alternans, right ventricular alternans, or biventricular alternans [3]. PA of the left cardiac system is commonly associated with aortic stenosis, hypertension, dilated or hypertrophic cardiomyopathy, and coronary heart disease. Conversely, PA of the right cardiac system often occurs with mitral stenosis, pulmonary heart disease, primary pulmonary hypertension, or right ventricular myocardial infarction. Although PA may be palpable in peripheral arteries (e.g., carotid or radial), it is typically detectable only in severe cases. While cardiac catheterization can directly detect and record ventricular and great artery pressures, elucidating ventricular systolic pressure disparities, its clinical use remains limited to its invasive nature.

Accurate assessment of cardiac function in PA patients is clinically essential. As a non-invasive imaging modality, echocardiography—including pulsed-wave Doppler and M-mode techniques—has emerged as the primary diagnostic tool for evaluating PA [4]. Previous studies have demonstrated that echocardiographic methods, particularly Doppler interrogation of the left ventricular outflow tract (LVOT) and M-mode analysis, can effectively identify PA characteristics [5]. However, due to the rarity of this clinical condition, the literature has been limited to isolated case reports or single-modality analyses, with no comprehensive studies employing multimodal echocardiographic approaches to systematically characterize this clinically significant phenomenon. This paucity of data has resulted in a notable gap in the literature regarding the integrated echocardiographic features of PA, especially their correlation with hemodynamic information. This study aims to fill this gap by retrospectively analysing multimodal echocardiographic imaging and corresponding clinical data from 16 patients with PA treated at our centre. Through this investigation, we aim to provide a systematic characterization of PA that enhances the understanding of its associated cardiac functional changes and hemodynamic consequences.

## Methods

### Study population

Sixteen patients with PA identified by echocardiography at our hospital between January 2021 and May 2025 were enrolled in this study. This retrospective study was approved by the local Institutional Ethics Committees in Clinical Research (KY2024-R250).

In this study, we employed pulse-wave Doppler echocardiography to characterize the non-invasive hemodynamic profile of PA. Beat-to-beat alternations of LVOT velocities with regular heart rhythms were considered to represent the PA phenomenon. The variation rate (∆) of the LVOT velocities between strong and weak beat were calculated using the following equation:

$$\begin{aligned}\Delta {\text{LVOT}} - {\text{VTI}} &={\text{ }}\left( {{\text{LVOT}} - {\text{VT}}{{\text{I}}_{{\text{strong beat}}}} - {\text{ LVOT}} - {\text{VT}}{{\text{I}}_{{\text{weak beat}}}}} \right) /{\text{ LVOT}}\\ & \qquad - {\text{VT}}{{\text{I}}_{{\text{strong beat}}}} \times {\text{1}}00\% \end{aligned}$$

Patients with a ∆LVOT-VTI exceeding 15% were included in our cohort.

### General clinical data

Standard clinical parameters, including age, gender, body mass index (BMI), blood pressure, New York Heart Association (NYHA), aetiology, presenting symptoms, electrocardiogram results, laboratory examinations, treatments administered, and follow-up assessments, were recorded for all subjects.

### Echocardiographic examination

Echocardiographic examinations were performed using either a GE Vivid E95 ultrasound system equipped with an M5S transducer (1.7–3.3 MHz) or PHILIPS EPIQ7 system with an S5-1 probe (1.5–3.5 MHz). Standard transthoracic echocardiography, including comprehensive M-mode, two-dimensional (2D) and Doppler assessments, was performed in all the study patients following the recommendations of the ASE guidelines. Dynamic 2D images (apical four-chamber, two-chamber, and long-axis views) were captured over 3 consecutive cardiac cycles and stored digitally for offline analysis.

LV inner diameter and aortic valve excursion were recorded by M-mode echocardiography. LVOT velocities and the flow of each valve orifice were assessed by pulsed-wave Doppler. The transmitral flow was displayed with colour M-mode Doppler. Mitral annulus velocities were derived from tissue Doppler imaging (TDI), and LV strain was quantified via speckle-tracking echocardiography. Simultaneously, bilateral carotid artery hemodynamics were recorded using Doppler interrogation.

### Conventional echocardiography parameters

Various parameters, including left atrial diameter (LAD), left ventricular end-diastolic dimension (LVDd), left ventricular end-systolic dimension (LVDs), interventricular septum thickness (IVST) and left ventricular posterior wall thickness (LVPWT), were measured using conventional echocardiography. Mitral inflow patterns were evaluated using Doppler echocardiography to measure peak early (E) and late (A) diastolic velocities.LV systolic(S′) and diastolic(E′ and A′) myocardial velocities were measured using pulsed-wave TDI. Additionally, hemodynamic evaluation included measurements of left and right ventricular outflow tract velocity-time integrals (LVOT-VTI and RVOT-VTI) by pulsed-wave Doppler. Left ventricular ejection fraction (LVEF) was calculated using the biplane Simpson’s method.

### Two-dimensional speckle-tracking echocardiography

High-frame-rate images were acquired in standard LV long-axis, four-chamber, and two-chamber views. For each view, three consecutive cardiac cycles were recorded in cine-loop format and stored for offline analysis using dedicated speckle-tracking software. The automated algorithm tracked myocardial motion to derive segmental longitudinal strain values generating corresponding bull’s-eye plots. Subsequent quantitative analysis included calculation of global longitudinal strain (GLS) and peak time standard deviation (PSD) across all LV segments.

### Statistical methods

SPSS 26.0 statistical software was used for data description and analysis. Continuous variables are presented as the mean ± standard deviation (SD) or median (interquartile range), as appropriate. Categorical variables are presented as frequency counts (n) and percentages (%), calculated as (number of cases) × 100. Data were tested for normality using the Shapiro-Wilk test prior to analysis.

Spearman correlation analysis was used to evaluate the correlation between non-invasive hemodynamic parameters (LVOT-VTI_strong beat_, LVOT-VTI_weak beat_, and ∆LVOT-VTI) and clinical data (such as NT-proBNP, hs-TNT, and outcomes) and myocardial mechanical parameters (GLS and PSD). Correlation coefficients with their corresponding p values are reported, with an r value of ± 0.1–0.3 considered weak, ± 0.3–0.5 moderate, and > ± 0.5 strong. A two-tailed p value < 0.05 was considered statistically significant. Correlations were visualized using scatter plots for continuous associations and box plots between continuous variables and categorical variables.

## Results

### Clinical data, treatment strategies, and prognosis of the PA patients

The clinical characteristics of the 16 patients are summarised in Tables 1 and 2. The cohort included patients aged 25 to 76 years (median age:40 years), with a male predominance (12 males, 4 females). The majority (13/16) were overweight. The primary symptoms reported were chest tightness and dyspnea (11/16), whereas cardiac auscultation revealed murmurs in 3 patients. Laboratory examinations revealed elevated N-terminal pro-B-type natriuretic peptide (NT-proBNP) in 15 patients(data unavailable for one patient), with a range of 163 to > 70,000 ng/L. Similarly, high-sensitivity cardiac troponin T (hs-TNT) levels were elevated in 13 patients, with a range of 16.5 to 224 ng/L.

**Table 1:** Clinical characteristics in 16 patients with pulsus alternans

Parameters	Patients (*n* = 16)
Age at diagnosis (years)	40 (25–76)^*^
*Gender*	
Male	12 (75.0%)
Female	4 (25.0%)
Follow-up (months)	21 (1–44)^*^
*BMI*	
< 25	3 (18.8%)
≥ 25	13 (81.3%)
*Hypertension*	
No	8 (50%)
Yes	8 (50%)
*NYHA*	
Ⅱ	3 (18.8%)
Ⅲ	7 (43.8%)
Ⅳ	6 (37.5%)
NT-proBNP(ng/L)	2679 (971–7621)^▲★^
hs-cTNT(ng/L)	43.8 (18.2-101.7)^▲^
*Etiology*	
DCM	7 (43.8%)
ICM	1 (6.3%)
ACM	1 (6.3%)
Valve replacement-related	2 (12.5%)
Valvular lesions-related	2 (12.5%)
Myocarditis	1 (6.3%)
Hypertensive cardiomyopathy	2 (12.5%)
*Outcomes*	
Good prognosis	11 (68.8%)
Poor prognosis	5 (31.2%)

*BMI* body mass index, *NYHA* New York Heart Association, *NT-proBNP* N-terminal pro-B-type natriuretic peptide, *hs-cTNT* high-sensitivity cardiac troponin T, *DCM* dilated cardiomyopathy, *ICM* ischemic cardiomyopathy, *ACM* alcoholic cardiomyopathy, *NA* not available

^*^Median (min, max); ^▲^Median (IQR); ^★^Given the presence of one extreme NT-proBNP value (>70,000 ng/L) exceeding 5 standard deviations from the median, analyses were performed both with and without truncation at 9398 ng/L (the 99th percentile of our cohort)

**Table 2:** Cardiac function classification, treatment strategies, and prognosis in 16 patients with pulsus alternans

Parameters	NYHA	Therapy	Follow-up (months)	Outcomes
Case 1	III	Calcium channel blockers, β-blocker	39	Serial follow-up
Case 2	II	ARBs, Aspirin	21	Serial follow-up
Case 3	II	Furosemide, spironolactone, ARBs	2	Rehospitalization
Case 4	IV	Furosemide	44	Serial follow-up
Case 5	II	Furosemide, antibiotics	39	Serial follow-up
Case 6	IV	Furosemide, spironolactone, trimetazidine	38	Serial follow-up
Case 7	III	β-blocker, ARBs, furosemide, spironolactone	32	Serial follow-up
Case 8	IV	Furosemide, spironolactone, ARBs, trimetazidine	32	Serial follow-up
Case 9	IV	Furosemide, ARBs, β-blocker	2	Heart transplantation
Case 10	III	Furosemide, spironolactone, ARBs	31	Serial follow-up
Case 11	III	Antibiotics, ARBs	6	Serial follow-up
Case 12	III	ARBs, β-blocker, spironolactone	20	Serial follow-up
Case 13	IV	Antibiotics, furosemide, lyophilized recombinant human brain natriuretic peptide	1	Death
Case 14	III	ARBs, β-blocker	1	Rehospitalization
Case 15	IV	Spironolactone, torsemide, β-blocker	7	Heart transplantation
Case 16	III	spironolactone, furosemide, β-blocker, ARBs	15	Serial follow-up

*NYHA* New York Heart Association, *ARBs* Angiotensin II receptor blockers

Underlying aetiologies included dilated cardiomyopathy (DCM, *n* = 7), ischemic cardiomyopathy (ICM, *n* = 1), alcoholic cardiomyopathy (ACM, *n* = 1), valve replacement-related cardiomyopathy (*n* = 2), valvular heart disease (*n* = 2), myocarditis (*n* = 1), and hypertensive cardiomyopathy (*n* = 2). Hypertension was present in 50% of the patients. Functional assessment according to the NYHA classification revealed class III (*n* = 7), class IV (*n* = 6), and class II (*n* = 3) heart failure. In our study, sinus tachycardia was the most predominant electrocardiogram (ECG) abnormality (10/16), followed by ST-T segment changes (8/16) and T-wave abnormalities (5/16).

Treatment adhered to heart failure guidelines, with diuretics and neurohormonal blockers (ARBs/β-blockers) achieving clinical stabilisation in most cases. Over a follow-up period of 1 to 44 months, adverse outcomes occurred in 5 patients: 2 underwent cardiac transplantation, 2 required rehospitalisation, and 1 died. The remaining 11 patients remain under active surveillance.

### Two-dimensional echocardiography characteristics

Left atrial enlargement was observed in 14 patients and was accompanied by varying degrees of LV enlargement. Ten patients exhibited ventricular wall thickening, while the remaining six exhibited normal wall thickness. LV systolic dysfunction was identified in 15 patients, with severity distributed as follows: severe (*n* = 10), moderate (*n* = 4), and mild (*n* = 1). Only one patient maintained normal LV systolic function (Table 3).

**Table 3:** Two-dimensional ultrasound characteristics in 16 patients with pulsus alternans

Parameters	LAD (mm)	LVDd (mm)	LVDs (mm)	IVST (mm)	LVPWT (mm)	LVEF (%)
Case 1	36	58	40	14	13	34
Case 2	33	44	30	13	12	32
Case 3	54	89	75	14	13	29
Case 4	41	73	63	9	9	18
Case 5	42	62	48	13	12	33
Case 6	52	65	57	11	11	16
Case 7	52	78	65	13	14	20
Case 8	48	75	65	10	10	18
Case 9	50	66	56	8	10	16
Case 10	43	61	52	10	10	22
Case 11	51	54	41	12	12	48
Case 12	45	59	50	12	11	37
Case 13	54	64	48	15	15	55
Case 14	48	71	59	10	10	24
Case 15	58	107	94	10	10	19
Case 16	49	67	61	11	11	22

*LAD* left atrial diameter, *LVDd* left ventricular end-diastolic dimension, *LVDs* left ventricular end-systolic dimension, *IVST* interventricular septum thickness, *LVPWT* left ventricular posterior wall thickness, *LVEF* left ventricular ejection fraction

### Myocardial mechanical ad hemodynamic characteristics

Three patients had biventricular PA, 13 had left ventricular PA, and none had right ventricular PA (Table 4).

**Table 4:** Myocardial mechanical and hemodynamic characteristics in 16 patients with pulsus alternans

Parameters	LVOT-VTI (strong beat) (cm)	LVOT-VTI (weak beat) (cm)	∆LVOT-VTI (%)	GLS (%)	PSD (ms)	Bilateral carotid artery alternans	Right ventricular alternans
Case 1	19.5	9.5	51	−9.6	95.0	Yes	None
Case 2	23.1	12.5	46	−8.2	57.0	NA	None
Case 3	27.3	22.1	19	−6.6	73.0	NA	None
Case 4	11.3	6.8	40	−1.2	81.0	Yes	None
Case 5	24.8	16.7	33	−7.0	79.8	NA	None
Case 6	15.8	10.0	37	−6.6	64.8	Yes	None
Case 7	14.0	8.4	40	−4.1	117.5	Yes	None
Case 8	14.2	10.2	28	−5.2	47.0	Yes	None
Case 9	12.8	9.5	26	−3.4	90.4	Yes	Yes
Case 10	15.1	10.7	29	−6.7	68.1	Yes	None
Case 11	29.2	14.1	52	−8.1	100.5	Yes	Yes
Case 12	24.6	17.3	30	−8.1	76.3	Yes	None
Case 13	26.4	14.5	45	−10.4	48.0	NA	None
Case 14	15.7	8.7	45	NA	NA	Yes	None
Case 15	15.4	11.6	25	NA	NA	Yes	None
Case 16	11.7	8.6	27	−3.8	71.5	Yes	Yes

*LVOT-VTI* left ventricular outflow velocity-time integral, *∆LVOT-VTI* variation rate between strong and weak beats of LVOT-VTI, *GLS* global longitudinal strain, *PSD* peak time standard deviation, *NA* not available

**Fig. 1:**
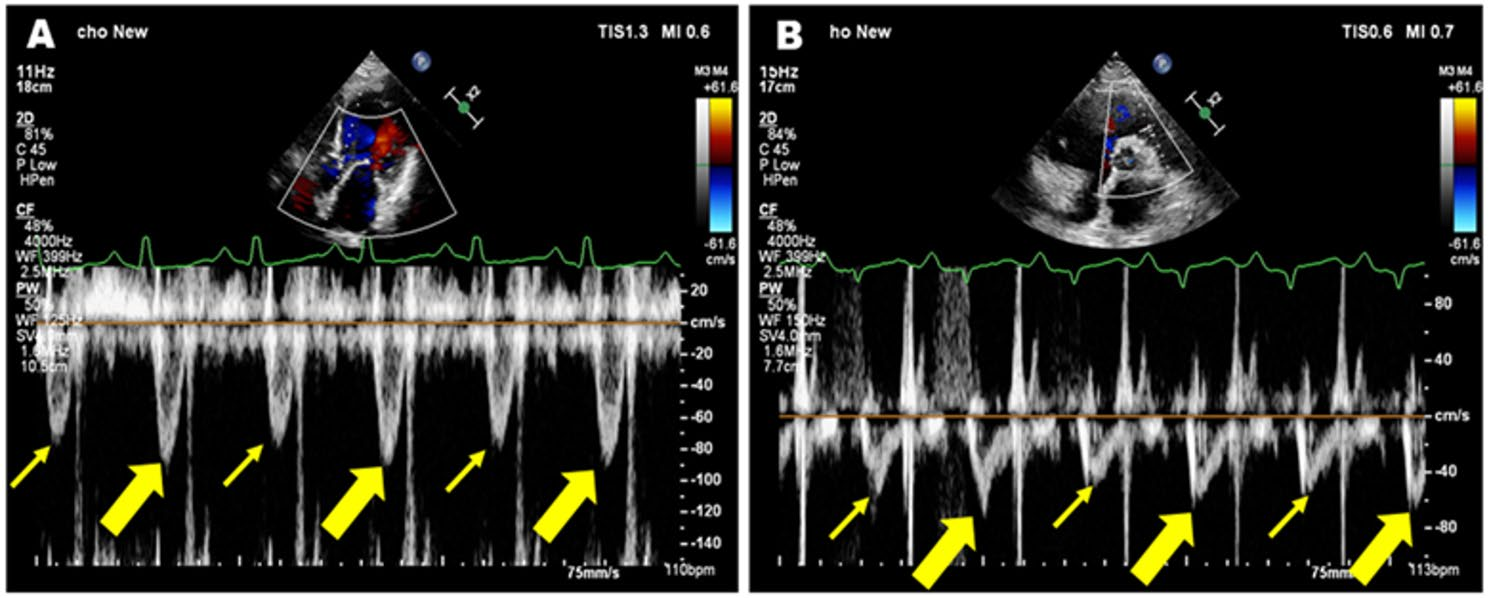
Pulsed-wave Doppler demonstrating a patient with biventricular PA. **A** The strong beat (bold yellow arrow) and the weak beat (thin yellow arrow) in the left ventricular outflow tract; **B** The strong beat (bold yellow arrow) and the weak beat (thin yellow arrow) in the right ventricular outflow tract

In biventricular PA, beat-to-beat alternations of LVOT velocities and right ventricular outflow tract velocities were displayed by pulse-wave Doppler (Fig. 1).

### Left ventricular pulsus alternans characteristics

**Fig. 2:**
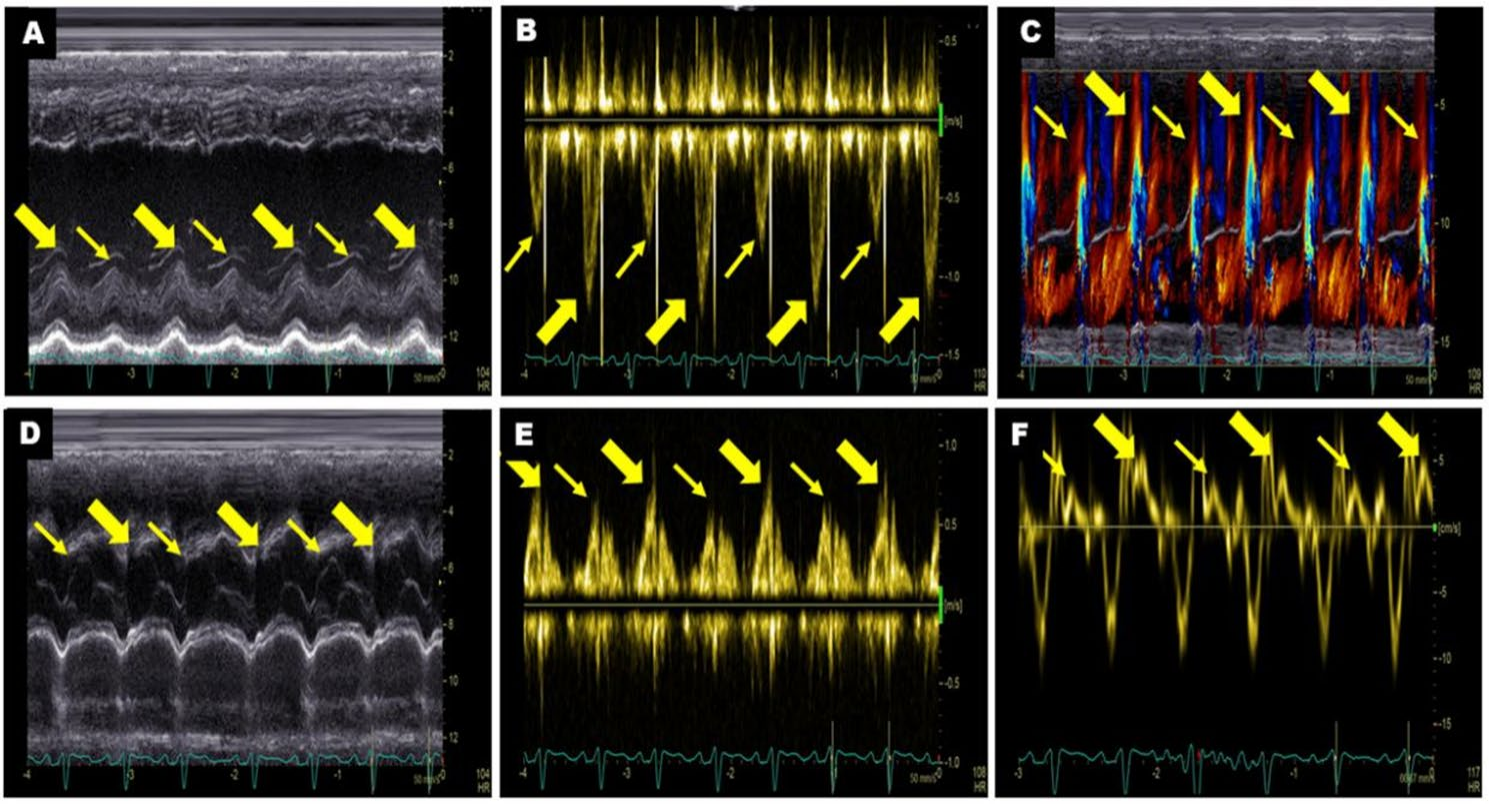
Multiple echocardiographic images of a patient with left ventricular pulsus alternans. **A** M-mode echocardiography showing beat-to-beat alternations in the left ventricular inner diameter. **B** Pulse-wave Doppler demonstrating beat-to-beat alternations of left ventricular outflow tract velocities. **C** Colour M-mode Doppler showing beat-to-beat alternations of diastolic transmitral flow. **D** M-mode echocardiography showing beat-to-beat alternations in aortic valve excursion during systole. **E** Pulse-wave Doppler showing beat-to-beat alternations of diastolic mitral valve flow velocities. **F** Tissue Doppler showing beat-to-beat alternations of mitral annulus systolic S′ velocities. The bold yellow arrow represents a strong beat, and the thin yellow arrow represents a weak beat on different echocardiographic images

Multiple echocardiographic methods revealed beat-to-beat alternations in both diastolic and systolic periods in all patients with left ventricular PA. Beat-to-beat alternations of left ventricular internal-diameter measurements (Fig. 2A) and aortic valve excursion (Fig. 2D) were demonstrated by M-mode echocardiography. Beat-to-beat alternations of LVOT velocities (Fig. 2B), diastolic mitral valve flow by pulse-wave Doppler (Fig. 2E), transmitral flow by colour M-mode Doppler (Fig. 2C) and mitral annulus systolic S’ velocities by tissue Doppler (Fig. 2F) were also displayed.

### Hemodynamic and mechanical characteristics of PA patients

**Fig. 3:**
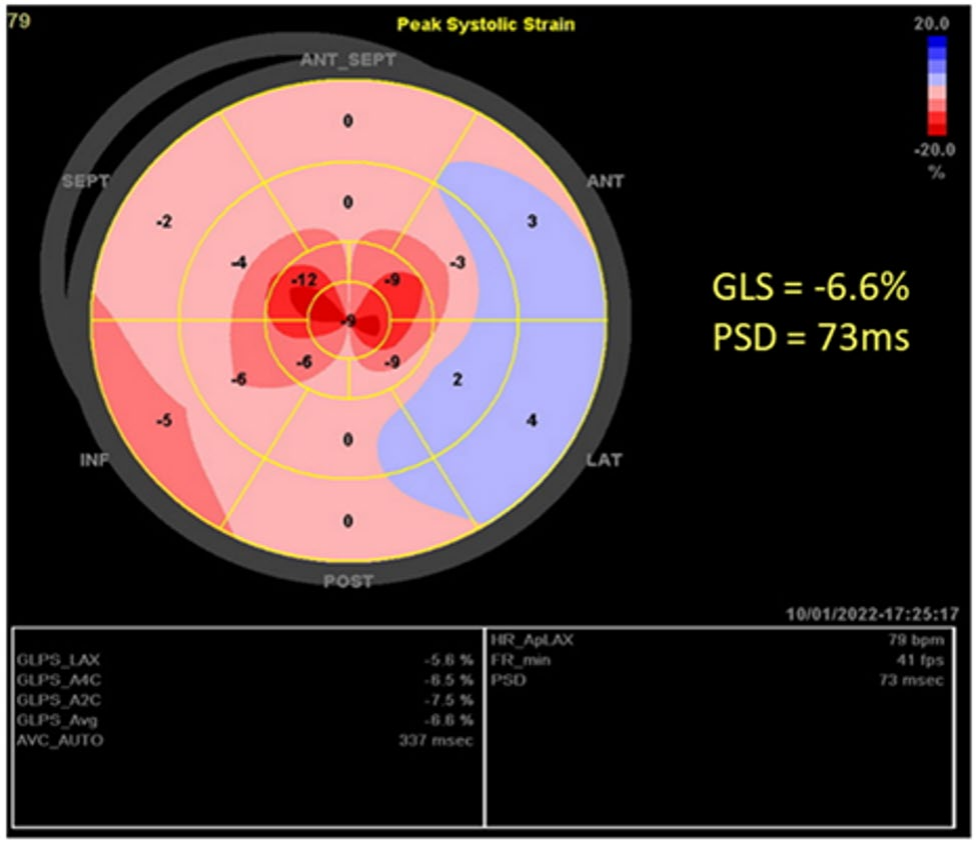
Bulls-eye map of left ventricular global longitudinal strain (GLS) in a patient with pulsus alternans. GLS decreased by −6.6% and PSD increased by 73ms. *GLS* global longitudinal strain, *PSD* peak time standard deviation

**Fig. 4:**
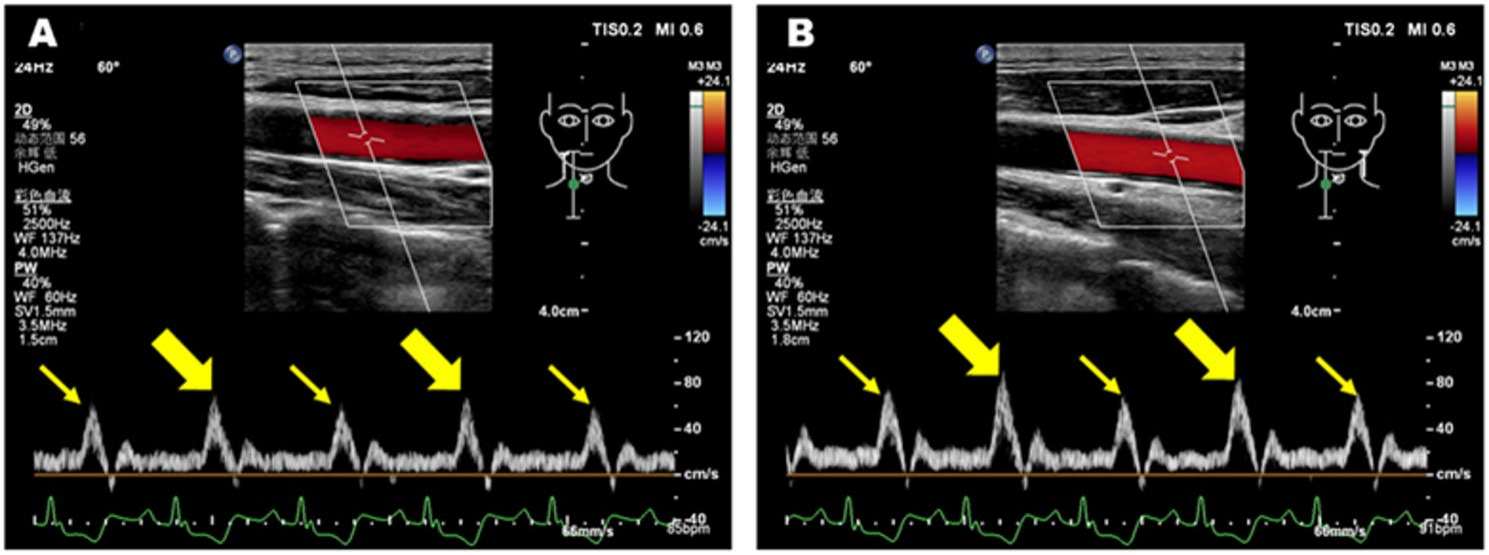
Doppler interrogation revealed marked beat-to-beat alterations in the bilateral carotid arteries of a patient with pulsus alternans. **A** Strong beat (bold yellow arrow) and weak beat (thin yellow arrow) of the right carotid artery. **B** Strong beat (bold yellow arrow) and weak beat (thin yellow arrow) of the left carotid artery

The LVOT-VTI_strong beat_ varied significantly, with a range of 11.3 cm to 29.2 cm, and the LVOT-VTI_weak beat_ also showed variation, ranging from 6.8 cm to 22.1 cm. The variation rate between strong and weak beats (∆LVOT-VTI) ranged of 19–52%, all exceeding the 15% threshold for PA in this cohort. Regarding the myocardial mechanical data, GLS decreased significantly in 14 patients, with a range of −1.2 to −10.4, and PSD increased, with a range of 47ms to 117.5ms (Fig. 3). Two patients were excluded from strain analysis because of suboptimal acoustic windows. Characteristic alternating flow patterns were detected in the bilateral carotid arteries of 12 patients (Fig. 4).

### Relationships between hemodynamic and myocardial mechanical parameters and clinical data

**Fig. 5:**
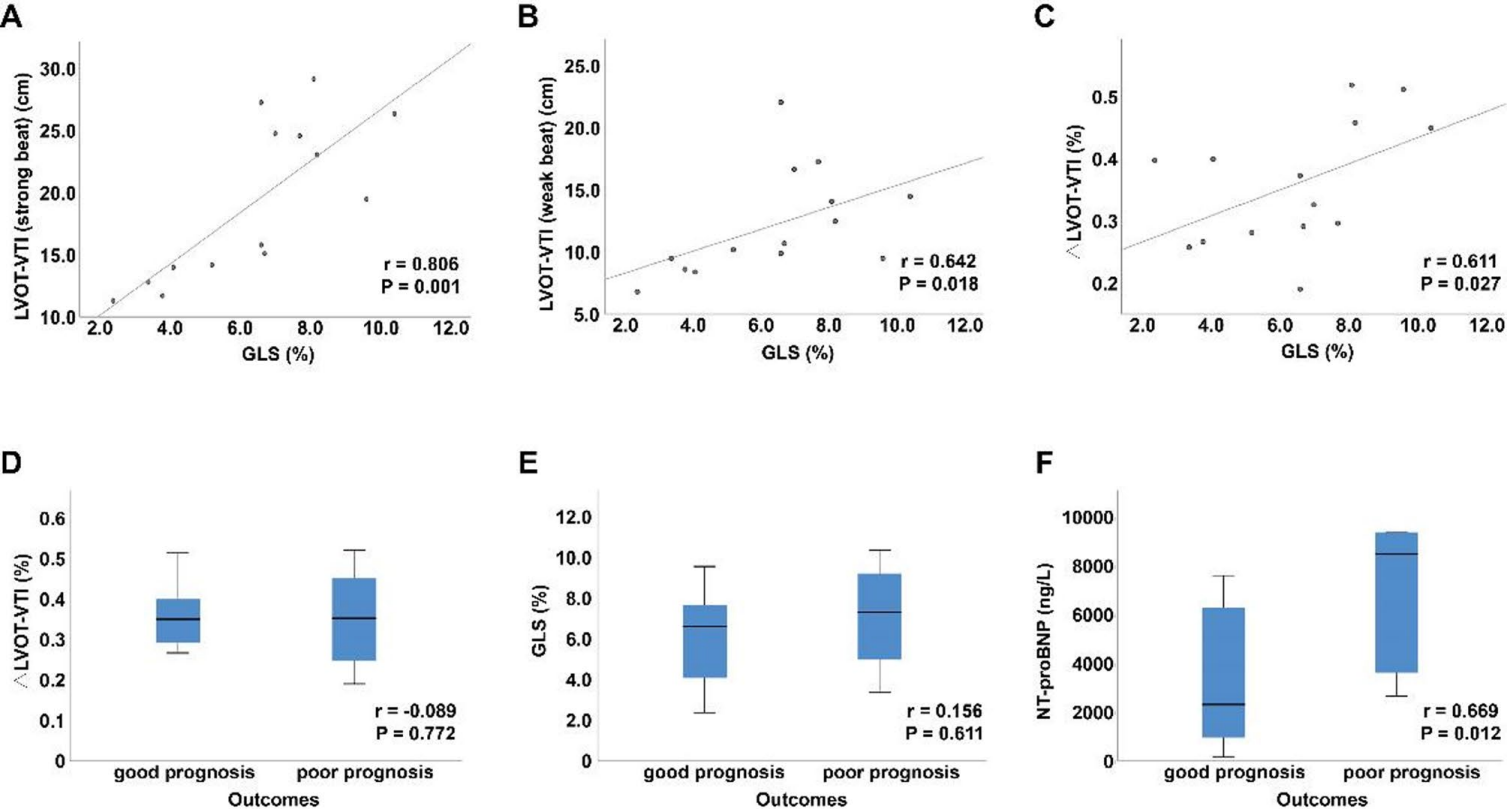
Correlations between hemodynamic data and GLS, ∆LVOT-VTI, GLS, NT-proBNP and outcomes in patients with pulsus alternans. **A** Scatter plot demonstrating a significant positive correlation between LVOT-VTI during strong beats and GLS (*r* = 0.806, *p* = 0.001). **B** Scatter plot demonstrating a significant positive correlation between LVOT-VTI during weak beats and GLS (*r* = 0.642, *p* = 0.018). **C** Scatter plot demonstrating a statistically significant positive correlation between ∆LVOT-VTI and GLS (*r* = 0.611, *p* = 0.027). **D** Box plot demonstrating a weak but non-significant negative correlation between ∆LVOT-VTI and poor prognosis (*r *= − 0.089, *p* = 0.772). **E** Box plot demonstrating a weak but non-significant positive correlation between GLS and poor prognosis (*r* = 0.156, *p* = 0.611). **F** Box plot analysis demonstrating a significant positive correlation between NT-proBNP levels and poor prognosis (*r* = 0.669, *p* = 0.012). *NT-proBNP* N-terminal pro-B-type natriuretic peptide, *LVOT-VTI* left ventricular outflow velocity-time integral, *∆LVOT-VTI* variation rate between strong and weak beats of LVOT-VTI, *GLS* global longitudinal strain

The relationships between non-invasive hemodynamic parameters and myocardial mechanical and clinical data in PA patients are shown in Table 5; Fig. 5. The results revealed strong correlations between hemodynamic profile (LVOT-VTI_strong beat_, LVOT-VTI_weak beat_ and ∆LVOT-VTI) and GLS in PA patients (*r* = 0.806, *P* = 0.001; *r* = 0.642, *P* = 0.018 and *r* = 0.611, *P* = 0.027, respectively). In this study, the relationships between hemodynamic, myocardial mechanical, clinical data and outcomes of PA patients were also analysed. NT-proBNP was significantly positive related to the outcomes of PA patients (*r* = 0.669, *P* = 0.012), whereas no correlation was detected between ∆LVOT-VTI or GLS and patient outcomes.

**Table 5:** Correlations of NT-proBNP, hemodynamic and myocardial mechanics parameters in pulsus alternans

Variable	*r*	*P* value
NT-proBNP (ng/L)/∆LVOT-VTI (%)	0.069	0.823
NT-proBNP (ng/L)/GLS (%)	− 0.022	0.943
NT-proBNP (ng/L)/PSD (ms)	0.008	0.979
**LVOT-VTI (strong beat) (cm)/GLS (%)**	**0.806**	**0.001**
LVOT-VTI (strong beat) (cm)/PSD (ms)	− 0.176	0.566
LVOT-VTI (strong beat) (cm)/outcomes	0.490	0.089
**LVOT-VTI (weak beat) (cm)/GLS (%)**	**0.642**	**0.018**
LVOT-VTI (weak beat) (cm)/PSD (ms)	− 0.382	0.197
LVOT-VTI (weak beat) (cm)/outcomes	0.468	0.106
**∆LVOT-VTI(%)/GLS (%)**	**0.611**	**0.027**
∆LVOT-VTI(%)/PSD (ms)	0.132	0.668
∆LVOT-VTI(%)/outcomes	− 0.089	0.772
GLS (%)/Outcomes	0.156	0.611
PSD (ms)/Outcomes	0.045	0.885
**NT-proBNP (ng/L)/outcomes**	**0.669**	**0.012**

*NT-proBNP* N-terminal pro-B-type natriuretic peptide, *LVOT-VTI* left ventricular outflow velocity-time integral, *∆LVOT-VTI* variation rate between strong and weak beats of LVOT-VTI, *GLS* global longitudinal strain, *PSD* peak time standard deviation; All the bold values indicate that these parameters have statistically significance (*P* < 0.05)

## Discussion

PA is an interesting clinical phenomenon characterized by an alternation of strong and weak ventricular contractions with a regular cardiac rhythm [6]. This finding typically indicates severe ventricular dysfunction and is associated with poor clinical outcomes [5]. Early recognition by clinicians is critical, as PA often serves as the initial manifestation of profound myocardial impairment. Echocardiography provides a first-line method for assessing cardiac functional changes and hemodynamic status continuously and noninvasively in PA. The present study contributes two key advances. First, we systematically integrate multiple echocardiographic imaging modalities to comprehensively characterize PA. Second, the combination of hemodynamic profiles and myocardial mechanical features was first investigated in this rare clinical entity by echocardiography, thereby providing novel insights into the pathophysiology of PA.

PA presents in three principal forms: left ventricular alternans (most common), biventricular PA (relatively rare) and right ventricular alternans (exceptionally uncommon). Cournand et al. [7] reported nine patients with biventricular PA, predominantly in patients with hypertension, arterioclerotic disease, biventricular heart failure, pulmonary hypertension, and right ventricular hypertension. Notably, compared to unilateral PA, biventricular PA is more strongly associated with congestive heart failure [12]. It is also often associated with coronary artery disease, particularly involving the left anterior descending coronary artery [8, 9]. In our cohort, three patients exhibited biventricular PA: two with hypertension and one normotensive individual. Of these, two had pulmonary hypertension combined with severe LV systolic dysfunction, while one had mild LV systolic dysfunction with normal pulmonary pressure. In this study, most PA patients presented with heart failure symptoms (chest tightness and dyspnea). NYHA functional classification was most commonly class III or class IV. However, not all patients exhibited obvious clinical manifestations, some showed no overt symptoms.

The precise pathophysiology underlying PA remains incompletely understood. Two primary mechanistic theories have been proposed:

(1)Hemodynamic theory: This explanation involves the Frank-Starling mechanism, where a strong contraction with increased stroke volume leads to more complete ventricular emptying and prolonged systole [10]. Consequently, the subsequent beat begins with reduced preload, resulting in weaker contraction and shorter diastolic filling. This alternating pattern of ventricular filling and emptying perpetuates the PA phenomenon. Even minor differences in the duration of weak and strong beats during contraction can result in significant differences in ventricular pressure at the onset of the next contraction, thereby impacting ventricular volume. However, the precise contribution of these factors to the amplitude of alternation remains to be further elucidated [2, 10].

(2)Cellular calcium cycling theory: Recent in vitro and molecular studies have demonstrated that mechanical alternans correlate with abnormalities in myocardial calcium handling [11]. During strong contractions, increased calcium release from the sarcoplasmic reticulum enhances contractility but depletes calcium stores, leading to weaker subsequent contractions. This cyclical pattern of calcium release and reuptake creates alternating strong and weak beats. This finding is also consistent with theories of imbalance between cardiac oxygen demand and oxygen availability [12, 13].

Echocardiography provides a continuous and noninvasive method for monitoring cardiac function and hemodynamic status. In patients with PA, ventricular contraction is characterised by a pattern of alternating strong and weak beats. Consequently, when evaluating SV and cardiac output (CO), the influence of these alternating beats, as well as mitral and aortic regurgitation, is pronounced. LVOT-VTI provides a practical, reproducible, and clinically accessible measurement that can effectively serve as an alternative marker for assessing cardiac function. It is independent of ventricular geometry and regional wall motion abnormalities, allowing for continuous tracking of SV and CO, thus making it valuable for hemodynamic monitoring in PA patients. Hence, LVOT-VTI demonstrates superior clinical utility for assessing cardiac function in patients with PA. However, owing to the special ventricular contraction of PA patients, neither the LVOT-VTI of the strong beat nor that of weak beat alone can adequately represent overall cardiac performance. To address this limitation, we propose a novel parameter, the variation rate of LVOT-VTI(∆LVOT-VTI), as a more comprehensive indicator of left ventricular systolic function in this population.

Two-dimensional speckle-tracking echocardiography allows quantitative analysis of the mechanical energy of longitudinal, radial, circumferential, and ventricular torsional motion of the myocardium without being constrained by the angle of the acoustic beam [14]. This technique reflects the degree of myocardial deformation and presents a novel approach for evaluating myocardial function and comprehensively understanding the mechanisms of cardiac mechanical motion [15]. While LVEF reflects global volume changes, myocardial strain analysis detects subtle alterations in regional and global contractility. In this study, we applied two-dimensional speckle-tracking echocardiography to patients with PA and measured GLS and PSD. Our findings indicate a statistically significant strong correlation between all the LVOT-VTI parameters of the hemodynamic profile and GLS in PA patients. The positive correlation suggests that higher LVOT-VTI values tend to be associated with greater GLS measurements. These findings indicate that LVOT-VTI may serve as one of several hemodynamic parameters influencing myocardial longitudinal function in PA, though its standalone predictive value remains constrained.

Furthermore, we observed consistent reductions in GLS across our cohort, including in patients with preserved LVEF (> 50%), except for two patients whose GLS and PSD data failed to be collected because of suboptimal imaging. Concurrently, all 14 subjects demonstrated elevated PSD values, indicating a characteristic pattern of both impaired myocardial contractility (reduced GLS) and significant mechanical dyssynchrony (increased PSD) in PA patients. The presence of PA fundamentally reflects severe impairment of cardiac contractile reserve. Persistently and significantly reduced LVOT-VTI directly quantifies the resulting severe forward flow insufficiency, which drives critical outcomes such as tissue hypoperfusion, multiorgan failure, and cardiogenic shock [16]. Concurrently, the absolute value of GLS serves as a quantitative measure of the profound exhaustion of global myocardial systolic functional reserve. A worse GLS indicates more extensive myocardial damage and a poorer prognosis. The integration of LVOT-VTI and GLS provides the most comprehensive assessment of the underlying pathophysiology in PA. This combination, particularly low LVOT-VTI with severely reduced GLS, is a powerful marker of extremely poor prognosis, necessitating aggressive intervention.

The treatments of PA patients vary depending on the underlying aetiology. For left ventricular PA, management strategies include hypotension management, diuretic therapy, or aortic valve replacement in selected cases [17]. For biventricular PA, consideration should be given to evaluating ischemic coronary artery disease. In our cohort, one patient underwent a heart transplant due to coronary artery disease and was followed up for an extended period. Another patient underwent heart transplantation because of dilated cardiomyopathy accompanied by severe cardiac insufficiency (LVEF 19%). Moreover, treatments for right ventricular alternans focus primarily on managing pulmonary embolism and lowering pulmonary artery pressure [5]. The majority of surviving patients in our study received long-term follow-up care following interventions such as diuresis, blood pressure reduction, ventricular function optimisation, or other appropriate therapies.

It has been suggested that there is a causal relationship between PA and sudden arrhythmic death. At the cardiomyocyte level, mechanical alternans may coincide with electrical alternans. Electrophysiological abnormalities, such as impaired impulse generation and conduction, are generally recognised as the underlying mechanisms contributing to electrical heterogeneity, instability, and sudden cardiac death [10]. Additionally, arrhythmias may arise through electromechanical feedback, where excitation-contraction coupling serves as a critical intermediary. Mechanical shortening or stretching of the myocardium can modulate key electrophysiological parameters [18, 19]. Thus, mechanical alternans may trigger electrophysiological disturbances via electromechanical feedback, potentially resulting in malignant arrhythmias and a poor prognosis. The prognosis of PA largely depends on the underlying aetiology, although it is generally regarded as an indicator. of poor prognosis. In our cohort, 5 of 16 patients (31.2%) experienced adverse outcomes: two underwent heart transplantation because of severe systolic dysfunction, two required rehospitalisation, and one passed away following rapid clinical deterioration after valve replacement. The remaining 11 patients had favourable outcomes, with follow-up durations exceeding six months (up to 44 months in some patients). Notably, one patient exhibited persistent PA at the six-month follow-up, but remained clinically stable with preserved cardiac function. Cardiac biomarkers (such as BNP and NT-proBNP) play an critical roles in the prognosis of heart failure [20, 21]. Our findings further support this, demonstrating a significant positive correlation between NT-proBNP levels and clinical outcomes in PA patients (*r* = 0.669, *P* = 0.012). Therefore, more clinical information should be taken into account when choosing the therapeutic method and assessing the prognosis of PA patients. In this cohort, a high proportion (31.2%) of PA patients had an adverse prognosis. This high rate of poor outcomes is primarily attributed to the study population predominantly comprising patients with severe heart failure (NYHA class III/IV), underscoring the challenges in managing PA effectively.

This investigation has several important limitations that should be acknowledged: (1) Limited sample size and population heterogeneity: The study cohort was relatively small (*n* = 16), and notably lacked cases of right ventricular alternans. Future studies with larger, more diverse patient populations are needed to strengthen the generalisability of the findings and enhance our understanding of PA across different clinical presentations. (2) Technical constraints in myocardial strain assessment: While two-dimensional speckle-tracking echocardiography provides valuable strain data, its inherent limitation of tracking myocardial motion in only two dimensions may result in incomplete speckle-tracking across myocardial layers, thereby impacting the accuracy of the result.

## Conclusion

Owing to the complexity of PA, accurately evaluating changes in cardiac function in this rare condition remains clinically challenging. In this study, we utilized multiple echocardiographic methods to delineate the characteristics of this interesting clinical phenomenon, which is suitable for the diagnosis of PA. Moreover, non-invasive pulse-wave Doppler parameters were introduced to elucidate the hemodynamic profile of PA patients, revealing the potential significance of these parameters in prognosis assessment. Additionally, the use of myocardial strain analysis as a novel method for evaluating myocardial mechanical features seems to be valuable in PA patients. Therefore, a comprehensive analysis using multimodal imaging is crucial for accurately identifying this disease, potentially improving the understanding of the pathophysiological mechanism of PA.

## Data Availability

The datasets used and/or analyzed during the current study are available from the corresponding author on reasonable requests.

## Abbreviations

PA: Pulsus alternans
GLS: Global longitudinal strain
PSD: Peak time standard deviation
NYHA: New York Heart Association
LVOT: Left ventricular outflow tract
LVEF: Left ventricular ejection fraction
VTI: Velocity time integral
